# A High-Quality Genome Assembly from Short and Long Reads for the Non-biting Midge *Chironomus riparius* (Diptera)

**DOI:** 10.1534/g3.119.400710

**Published:** 2020-02-14

**Authors:** Hanno Schmidt, Sören Lukas Hellmann, Ann-Marie Waldvogel, Barbara Feldmeyer, Thomas Hankeln, Markus Pfenninger

**Affiliations:** * Molecular Ecology Group, Senckenberg Biodiversity and Climate Research Centre, Frankfurt am Main, Hessen, Germany; † Vector Genetics Laboratory, Department of Pathology, Microbiology and Immunology, School of Veterinary Medicine, University of California Davis, CA; ‡ Molecular Genetics and Genome Analysis Group, Institute of Organismic and Molecular Evolutionary Biology, Johannes Gutenberg-University, Mainz, Germany; § LOEWE Centre for Translational Biodiversity Genomics (LOEWE-TBG), Frankfurt, Germany

**Keywords:** hybrid genome assembly, *Chironomus riparius*, recombination rate

## Abstract

*Chironomus riparius* is of great importance as a study species in various fields like ecotoxicology, molecular genetics, developmental biology and ecology. However, only a fragmented draft genome exists to date, hindering the recent rush of population genomic studies in this species. Making use of 50 NGS datasets, we present a hybrid genome assembly from short and long sequence reads that make *C. riparius*’ genome one of the most contiguous Dipteran genomes published, the first complete mitochondrial genome of the species, and the respective recombination rate among the first insect recombination rates at all. The genome assembly and associated resources will be highly valuable to the broad community working with dipterans in general and chironomids in particular. The estimated recombination rate will help evolutionary biologists gaining a better understanding of commonalities and differences of genomic patterns in insects.

Non-biting midges (Chironomidae) are dipterans like the model organisms *Drosophila* fruit flies and *Anopheles* mosquitoes. The species *Chironomus riparius* (synonym *C. thummi* or *C. thummi thummi*) is particularly important in ecotoxicological (Williams *et al.* 1986; Lee *et al.* 2009), molecular genetic (Hankeln *et al.* 1997; Hankeln and Schmidt 1990), developmental (Klomp *et al.* 2015) and ecological (Armitage *et al.* 1995; Rosenberg 1992; Foucault *et al.* 2019) research. Recently, *C. riparius* has also emerged as a promising organism for transcriptomic (Schmidt *et al.* 2013; Nair *et al.* 2011; Marinković *et al.* 2012) and genomic studies (Oppold *et al.* 2017; Waldvogel *et al.* 2018). Although important population genomic parameters are already available for *C. riparius* (*e.g.*, the mutation rate *μ*; (Oppold and Pfenninger 2017)), analyses still rely on a fragmented Illumina-only genome assembly (Oppold *et al.* 2017). Here we present a high-quality hybrid genome assembly from short and long reads, along with an estimate for the species-specific recombination rate, the first complete mitochondrial genome for this species and a reference transcriptome based on several life stages. This is an important step forward to enable more complex genomic studies on *C. riparius* and hence understand variability in dipteran genome evolution patterns.

## Materials and Methods

### Assembly strategy

The assembly of the Illumina-PacBio-hybrid genome consisted of five major steps: (1) De Bruijn graph assembly of the Illumina reads, (2) hybrid assembly of Illumina contigs and raw PacBio reads, (3) error correction of hybrid contigs by mapping of Illumina data, (4) scaffolding of the contigs using mate-pair reads, (5) closing the remaining gaps with corrected PacBio and Illumina paired end sequences.

### Samples and PacBio sequencing

Long reads (Supplementary Table S1, dataset 01) were sequenced from 52 female imagines that originated from one egg clutch of a strict inbred line (described in (Oppold and Pfenninger 2017)) of the same *C. riparius* laboratory culture that has been used for previous draft genome sequencing (Oppold *et al.* 2017). DNA was isolated with the QIAGEN Gentra Puregene Tissue Kit according to manufacturer’s instructions and sequenced on six SMRT Cells on a Pacific Biosciences RS II machine.

### Genome assembly

Illumina data (Supplementary Table S1, datasets 02-06) was sequenced from approximately 50 larvae from a long-standing laboratory culture (Oppold *et al.* 2017). Quality processing of the reads was done using Trimmomatic v0.32 (Bolger *et al.* 2014) with default parameters and FastQC v0.11.3 (Andrews 2010). Additionally, we filtered out mitochondrial reads using BBDuk from the tool package BBMap v35.85 (Bushnell 2014) with k = 41 and hdist = 2.

We assembled the quality processed Illumina reads using the De Bruijn graph assembler Platanus v1.2.4 (Kajitani *et al.* 2014) with kmer-sizes between k = 32 and k = 84 and s = 6. The resulting contigs plus the raw PacBio reads were then used as input for the program DBG2OLC v1.0 (Ye *et al.* 2016) and assembled with recommended settings (k = 17, KmerCovTh = 2, MinOverlap = 20, AdaptiveTh = 0.002). The assembly was screened by BLASTN searches (Blast v2.3.0+ (Altschul *et al.* 1990)) for sequences originating from the common bacterial endosymbiont *Wolbachia* (Correa and Ballard 2016) and five contigs were removed, thereby getting rid of all *Wolbachia* contaminations. Since the raw PacBio reads were used for assembly to achieve highest contiguity of contig sequences, we subsequently used proovread v2.13.12 (Hackl *et al.* 2014) to correct the DBG2OLC contig sequences iteratively. In the first pass, we used the Platanus contig sequences described above, and in a second pass the additional Illumina reads (100x coverage; Supplementary Table S1, datasets 07-11). The Illumina data for error correction was sequenced from progeny of the same one egg clutch as the larvae for the PacBio sequences to allow for highest sequence conformity and thus correction confidence. Since hybrid assembly is a highly complex procedure and we did not want to miss any information, we screened Illumina-only and PacBio-only assemblies for additional sequence information lacking in the DBG2OLC contigs. The Platanus-derived contigs described above were compared to the DBG2OLC contigs by BLASTN searches with perc_identity = 80. The PacBio reads were assembled with Canu v1.0 (Berlin *et al.* 2015) using default settings and the output contigs (Supplementary Table S2) used for BLASTN searches as described for the Platanus contigs. All contigs from both approaches that did not match DBG2OLC contigs with at least 80% were then added to the DBG2OLC assembly. These sequences were then scaffolded using SSPACE v3.0 (Boetzer *et al.* 2011) with x = 0, n = 25 and mate-pair libraries with 3 and 5.5 kb insert size (S.D. 0.8). Scaffold gaps were addressed with an iterative gap closure process. First, we corrected PacBio raw reads with Illumina reads by proovread applying default settings, and then used them to close gaps applying PBJelly v15.2.20 (English *et al.* 2012) with default settings. Afterward, datasets 02-06 (Supplementary Table S1) were used as input to five iterative rounds of GapFiller v1.10 (Boetzer and Pirovano 2012) with default parameters and average insert size with insert size variation.

### Estimation of the recombination rate

Recombination rate estimates (*ρ*) were derived from 20 field isolates from five European natural populations (Supplementary Table S1, datasets 31-50) by applying a *reversible jump Markov Chain Monte Carlo mechanism* (rjMCMC) implemented in the program LDhelmet v1.7 (Chan *et al.* 2012) individually for each scaffold. LDhelmet is a derivative of LDhat (Auton *et al.* 2012), especially modified to fit genomic characteristics that differ from hominids to *Drosophila* (for example higher SNP density). Since we anticipate similar patterns in *Chironomus*, we chose LDhelmet and mainly followed the parameter recommendations of the authors. The ultimate LDhelmet analysis with the *rjmcmc* command was run for each scaffold with a block penalty of 50.0 (as recommended; parameter of negligible influence on results (Smukowski Heil *et al.* 2015)) and a window size of 50 SNPs (as in the data preparation). We used a burn-in of 1,000,000 iterations and subsequently ran the Markov chain for 10,000,000 iterations (see Supplementary Methods S1 for details).

Using ancestral linkage disequilibrium-based methods for the estimation of recombination rates heavily profits from a genetic map provided at the stage of phasing the SNP data. Since there is no such a resource for *C. riparius*, a constant rate was used. Although this is the default of the phasing algorithm applied, it may introduce a bias into the estimation of *ρ* based on this data.

### Genome annotation

The annotation of gene content along the genome was aided by construction of a reference transcriptome, which assembled from 19 cDNA sequence data sets (Supplementary Table S1, datasets 12-30). We used Illumina and 454 Roche sequence reads from embryos, larvae and adults (both sexes; treated and untreated; see Supplementary Table S1 and references therein for details) to reach a maximum of expressed genes in order to optimize gene annotation. First, data sets were pre-processed using fastqc (Andrews 2010) and BBDuk from the BBMap package v35.85 (Bushnell 2014). Thereby, sequence adapters were trimmed using k = 23, mink = 11, hdist = 1, tbo and tpe options. 3′ bases with phred quality below 20 were trimmed and reads with average phred quality below 20 discarded. Assembly of the cleaned reads was then performed in two separate steps for Illumina and 454 data with Trinity v2.3.2 (Grabherr *et al.* 2011) using uneven k-mer sizes from 25 to 31. The best assembly was identified to be with k = 25 using assembly metrics like N50 and a search for core orthologous genes with BUSCO v1.2b (Simão *et al.* 2015) and used further on. The resulting assemblies for Illumina and 454 Roche data, respectively, were then merged and duplicate contigs removed using dedupe from the BBmap package with mid = 90. The resulting final transcriptome assembly was then used for gene annotation.

To ensure discovery of most repeat sequences in the draft genome, we extended the custom repeat library from (Oppold *et al.* 2017) with repeat sequences extracted manually from the draft genome presented in this study.

The whole annotation process was performed with the MAKER2 v2.31.8 (Cantarel *et al.* 2008; Holt and Yandell 2011) pipeline and affiliated programs. We used a three-round iterative process with the reference transcriptome and repeat library described above, three *C. riparius*-specific gene models and the SwissProt database as input (see Supplementary Methods S2 for details).

### Assembly and annotation of the mitochondrial genome

The reconstruction of the mitochondrial genome sequence was performed using the program MITObim v1.8 (Hahn *et al.* 2013) on the large paired end dataset 03. MITObim applies a baiting and iterative mapping approach to short read data. Using a mitochondrial reference sequence (here an unpublished, partial Sanger sequence of *C. riparius*’ mitochondrial genome), the program performs a mapping to gather all reads belonging to the mitochondrial genome and assembles them with MIRA v4.0.2 (Chevreux *et al.* 1999) in the first round. Then it uses the produced sequence to again fish for mitochondrial reads for further assembly. This is repeated until the number of mapped reads becomes stationary. MITObim was run four times with the reference sequences of *C. riparius* being modified in length to allow for different starting points of the procedure. All four output sequences were then aligned in MEGA v7.0.7 (Kumar *et al.* 2016) and manually integrated into a consensus sequence. This consensus sequence was annotated by MITOS WebServer (Bernt *et al.* 2013) using the genetic code *05 - invertebrate*. The whole sequence and all annotations were finally checked and, where necessary, corrected manually.

### Data availability

Raw sequence reads are available through NCBI Sequence Read Archive, accession numbers are detailed in the Supplementary Table S1. Project number for the genome assembly is PRJEB27753 and for the mitochondrial genome assembly PRJEB27747. Supplemental material available at figshare: https://doi.org/10.25387/g3.11575059.

## Results and Discussion

### Genome assembly

**Figure 1:**
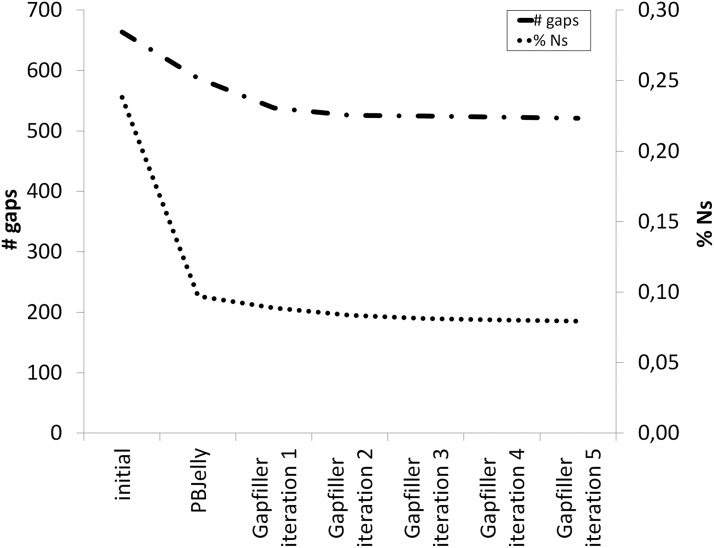
Effect of gap filling procedures. Gap filling with corrected PacBio reads (PBJelly) and Illumina paired end reads (Gapfiller). Shown is the decrease in number of gaps (“# gaps”, dashed line) and fraction of undefined nucleotides (“% Ns”, dotted line) in the scaffolds during the iterative gap filling process.

The 1,155,855 PacBio reads had an average length of 4,751 bp, and the longest read was 48,745 bp. The final hybrid assembly consisted of 752 scaffold sequences with a total length of 178,167,951 bp (Table 1). The total assembly length fits the published genome size of ∼200 Mb estimated by flow cytometry (Schmidt-Ott *et al.* 2009), given that regions of low sequence complexity (*e.g.*, highly repetitive parts of centromeres and telomeres) are likely not to be resolved and thus missing. In light of the many tandem-repetitive element clusters interspersed in the genome of *C. riparius* (Schmidt 1984; Hankeln *et al.* 1994) it is therefore reasonable to assume that scaffold ends represent borders to internally repetitive heterochromatic regions in most cases. The N50 of 539,778 bp of the current genome draft is almost twice as high as for a previous version (Table 1). Gap filling drastically reduced the final unresolved base content (N’s) of the assembly down to 0.08%, with the PacBio reads being especially helpful (Figure 1). On average 96.6% of the Illumina sequence reads could be mapped back to the draft genome (Supplementary Table S3), corroborating our assumption that only highly repetitive areas are underrepresented in the genome draft.

**Table 1:** *– Characteristics of* C. riparius *genome assembly*. Shown are the improvements in quality by combining short and long reads in comparison to the previous Illumina-only assembly. Values are based on the nuclear genome only

	Illumina-only draft genome (Oppold *et al.* 2017)	Hybrid assembly (present study)	Degree of improvement
number of scaffolds	5,292	752	1/7
total scaffold length (bp)	180,652,019	178,167,951	equal
average scaffold length (bp)	34,136	236,926	x 7
longest sequence (bp)	2,056,324	2,626,431	+25%
N50	272,065	539,778	x 2
N content (%)	15.96	0.08	1/200
BUSCOs found (complete and fragmented)	92.8	93.7	+ 1%

### Recombination rate

Mean *ρ* values (always given per base pair within 50 kb windows) in *C. riparius* ranged between 0.04 and 0.07, thus lying within the range of those estimated for *Drosophila melanogaster* (0.01 to 0.11; (Chan *et al.* 2012)).

**Figure 2:**
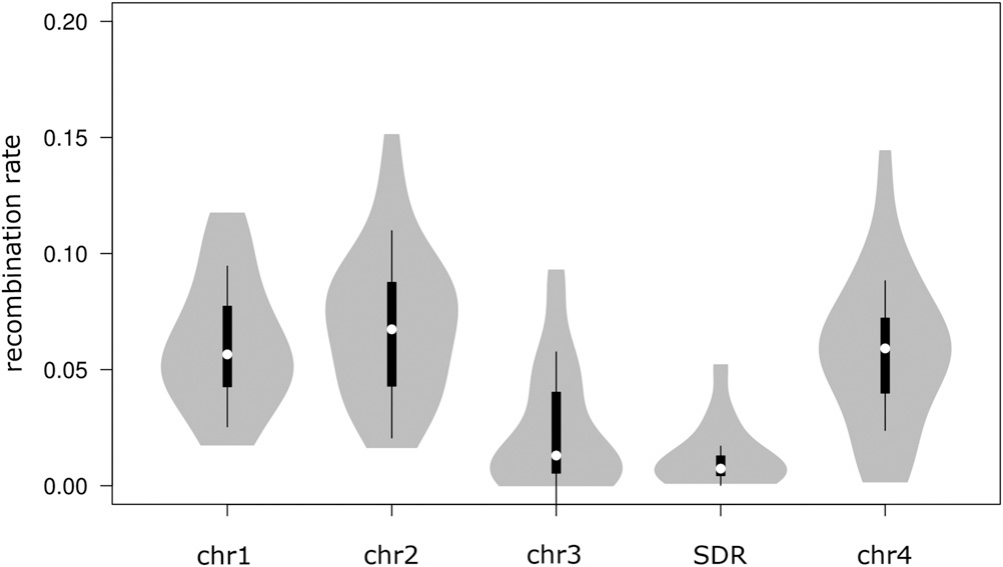
*C. riparius* chromosome-specific recombination rates. Recombination rates from all individuals across populations were pooled. Chromosome 3 is represented without the identified part of the sex determining region, which is displayed separately (SDR). White circles show medians, box limits indicate the 25^th^ and 75^th^ percentiles, whiskers extend 1.5 times the interquartile range from the 25^th^ and 75^th^ percentiles, polygons represent density estimates of data and extend to extreme values. Kruskal-Wallis test (nonparametric for data without normal distribution) with Dunn’s multiple comparison post-test (GraphPad Prism v5) revealed a significant difference (*P* < 0.001) between chromosome 3 as well as the SDR relative to all other chromosomes. Chr = Chromosome.

Recombination should be less frequent across sex-determining regions, because reciprocal exchange of chromosomal parts is only possible in the germ line of female individuals (Wright *et al.* 2016) (but see Rodrigues *et al.* 2018). *C. riparius* has a sex-determination system with heterogametic males, bearing a sex-determining region (SDR) on chromosome 3 that is being interpreted as an emerging sex chromosome (Kraemer and Schmidt 1993; Michailova *et al.* 2009). We identified the candidate SDR-containing scaffold by BLASTN searches with the sequence of the single copy gene *CpY* (NCBI accession number X82317.1) as query. Indeed, the SDR scaffold (# 549) showed a large region with lowered recombination rates around *CpY* (Supplementary Figure S1). When extracting the last 600,000 bp from this scaffold, we observed a mean *ρ* of 0.014 compared to overall estimates between 0.024 and 0.07 for the four chromosomes (Figure 2). This seems reasonable since recombination should be roughly halved in the SDR. Interestingly, the remaining parts of chromosome 3 without the identified part of the SDR also have relatively low recombination rates compared to all other chromosomes (Figure 2; Kruskal-Wallis test, *P* < 0.001), potentially due to further fragments of the SDR being present along this chromosome or impacts of the SDR on the genomic surroundings.

### Annotation

13,449 protein-coding genes were annotated across the *C. riparius* nuclear genome (Table 2). There is a slight negative correlation between number of genes and number of exons per gene across chironomid genomes (Supplementary Figure S2). This may point toward multi-exonic genes being split up into several genes in draft genomes with large numbers of annotated genes. The relatively high number of exons per gene (5.1) in a relatively low number of genes in the *C. riparius* genome annotation compared to other chironomid genomes therefore suggest a high quality due to the assembly’s contiguity. Applying the algorithm BUSCO v1.2b (Simão *et al.* 2015), we found orthologous sequences to 93.7% of arthropod core genes (Table 1). Therefore, we can assume the genome to be almost complete also in terms of gene space.

**Table 2:** *– Comparative statistics of the nuclear genome’s annotation*. Content of protein-coding genes in the genome of *C. riparius* compared to published genomes of other chironomids and *Drosophila melanogaster*

	gene count	average number of exons per gene	average exon length (bp)	protein coding part of the genome (%)
*Chironomus riparius* (this study)	13,449	5.1	378	14.6
*Chironomus tentans* (Kutsenko *et al.* 2014)	15,120	3.8	312	9
*Polypedilum vanderplanki* (Gusev *et al.* 2014)	17,137	4.3	324	20.2
*Polypedilum nubifer* (Gusev *et al.* 2014)	16,553	4.0	328	20.3
*Belgica antarctica* (Kim *et al.* 2017; Kelley *et al.* 2014)	11,005	5.0	321	19.6
*Clunio marinus* (Kaiser *et al.* 2016)	14,041	4.5	329	29.6
*Parochlus steinenii* (Kim *et al.* 2017)	13,468	6.2	215	13.0
*Drosophila melanogaster* (Adams *et al.* 2000)	13,907	5.5	538	18.3

**Figure 3:**
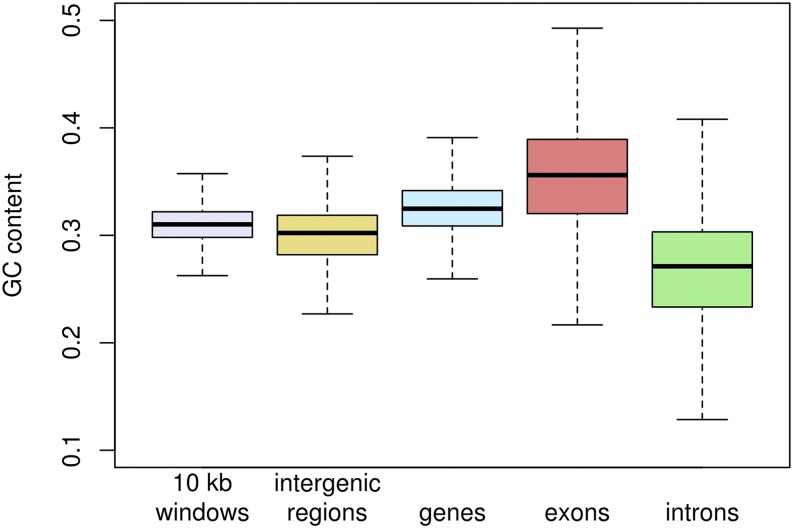
GC content for genomic features. Different genomic features revealed differences in GC content. GC content in exons resided above genome average, while the opposite was found for introns. 10 kb windows were generated without regard to their content. Box limits indicate the 25^th^ and 75^th^ percentiles, whiskers extend 1.5 times the interquartile range from the 25^th^ and 75^th^ percentiles.

The proportionate genome-wide GC content in *C. riparius* is 0.311, which is close to the average of 0.332 across chironomid genomes, including *C. tentans* (0.312), *Polypedilum vanderplanki* (0.28), *P. nubifer* (0.39), *Belgica antarctica* (0.39), *Clunio marinus* (0.317) and *Parochlus steinenii* (0.322). Average GC content in chironomids is thus at the lower end compared to other insect genomes (Samanta 2007). Across a broad phylogenetic range including plants, invertebrates and vertebrates, GC content has been shown to be higher in exons than introns and might have evolved as a determinant of exon selection (Amit *et al.* 2012). To assess this for the *C. riparius* genome, we inferred GC content for all non-overlapping 10 kb windows throughout the genome assembly (N = 17,566), all coherent regions without genes (N = 24,771), all protein-coding genes (N = 13,449), all exons (N = 68,943) and all introns (N = 54,860). GC content across the random 10 kb windows was on average 0.310 +/− 0.026 (mean +/− s.d.), perfectly mirroring the GC content of 0.311 for the whole genome assembly. Windows containing genes had a slightly higher GC content than genome average (0.327 +/− 0.032) and windows without genes had a slightly lower GC content than genome average (0.299 +/− 0.044). This difference was much more pronounced between exons (0.355 +/− 0.061) and introns (0.269 +/− 0.056), with exons being the feature with the highest and introns with the lowest GC content (Figure 3). The differences between categories were highly significant (*P* < 0.0001) for all pairwise comparisons applying Mann-Whitney tests with Bonferroni correction.

101,693 regions of up to 30 kb in length were annotated as repetitive sequences (9.14%). Compared to the Illumina-only genome draft (Oppold *et al.* 2017), the inclusion of long sequence reads has significantly increased detection of repeats by 41%. Given the heavy load of repetitive sequences in the *C. riparius* genome (Schaefer and Schmidt 1981), however, this value most likely still underestimates the true repeat content due to unresolved large heterochromatic regions.

**Figure 4:**
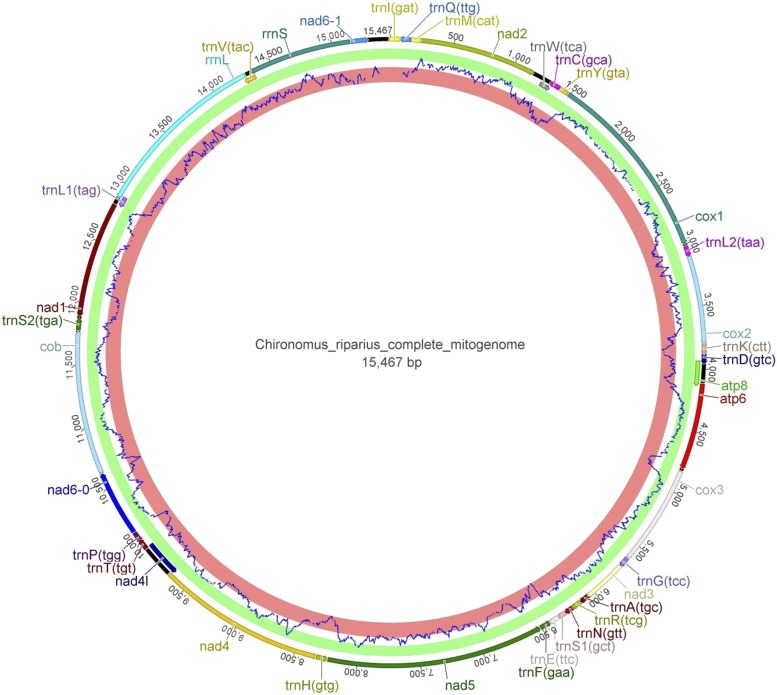
Mitochondrial genome of *C. riparius*. The circular genome consists of 15,467 bp. Prediction of protein-coding sequences by the EMBOSS tool tcode (blue graph at the inner edge of the genome; green ring = coding, red ring = non-coding) mainly is consistent with the annotation from MITOS.

The mitochondrial genome’s length is 15,467 bp, which is in line with other dipteran values. All 37 genes of the mitochondrial genome could be annotated (Figure 4, Supplementary Table S4). Gene order follows the one conserved across Diptera (with the exception of Culicidae having the *trnA* and *trnR* genes switched (Behura *et al.* 2011; Hao *et al.* 2017)), sharing complete synteny even with drosophilids (Montooth *et al.* 2009) from which they split an estimated 250-300 Myr ago (Cranston *et al.* 2012; Bolshakov *et al.* 2002).
